# Dyslexia and cognitive impairment in adult patients with myotonic dystrophy type 1: a clinical prospective analysis

**DOI:** 10.1007/s00415-020-10161-6

**Published:** 2020-08-26

**Authors:** K. Gutschmidt, S. Wenninger, F. Montagnese, B. Schoser

**Affiliations:** Department of Neurology, Friedrich-Baur-Institute, Ludwig-Maximilians-University Munich, Ziemssenstr. 1a, 80336 Munich, Germany

**Keywords:** Dyslexia, Reading disorder, IQ, Cognitive impairment, Neurodegenerative

## Abstract

**Background:**

Cognitive impairments in patients with myotonic dystrophy type 1 (DM1) have often been described, however, there are only few studies differentiating between partial performance disorders and mental retardation in common. This study focused on the evaluation of reading performance and the frequency of dyslexia in adult DM1 patients.

**Methods:**

We performed a prospective cohort study including genetically confirmed adult DM1 patients registered in the DM registry of Germany or the internal database of the Friedrich-Baur-Institute, Munich, Germany. For the assessment of the patients’ reading and spelling performance, we used the standardized and validated test ‚Salzburger Lese- und Rechtschreibtest‘ (SLRT II). The ‚CFT-20 R Grundintelligenztest Skala 2‘ in revised ("R") version (CFT 20-R), determining the intelligence level, was appropriate to differentiate between dyslexia and general mental retardation. The diagnosis of dyslexia, the combined reading and spelling disorder, was based on the guidelines for diagnosis and therapy of children and adolescents with dyslexia 2015 (S3-guideline) providing (1) the criterion of the divergence from age level and (2) the criterion of IQ-divergence.

**Results:**

Fifty-seven DM1 patients participated in our study. Evaluating the reading performance, 16 patients fulfilled the divergence criteria of the age level and 2 patients the IQ-divergence criteria. In total, the diagnosis of a reading disorder was given in 18 DM1 patients (31.6 %). In 11 out of these 18 patients with a reading disorder, a relevant impairment of spelling performance was observed with at least three spelling errors. As there are no normative values for adults in spelling performance, we assume a combined reading disorder and dyslexia, in those 11 DM1 patients (19.3 %). Regarding the separate analyses of the test procedures, in the SLRT II the performance was below average in 40.4 % of all patients for ‘word reading’ and in 61.4 % of all patients for ‘pseudoword reading’. There was a significant positive correlation between the CTG expansion size and a reading disorder (p=0.027). The average IQ of 17 examined DM1 patients was in the lower normal range (86.1 ± 19.1). 54.5 % of patients with reading disorder had a normal IQ.

**Conclusion:**

The calculated prevalence of dyslexia in the DM1 study cohort was 19.3 % and thus considerably increased compared to the normal German population. As dyslexia is not equivalent to a general cognitive impairment, it is important not to miss dyslexic features in cognitive inconspicuous DM1 patients. Case-by-case one should consider a differential diagnostic approach, as individualized therapies can be offered to support dyslexic patients in their performance.

## Introduction

With an approximate prevalence of 10–20 to 100,000 people, myotonic dystrophy type 1 (DM1) is one of the most frequent inherited neuromuscular diseases in adulthood [1, 2]. This multisystemic disorder is caused by a genetic mutation of the dystrophia myotonica protein kinase (DMPK) gene on chromosome 19q13.3, overexpressing the CTG triplet with at least 50 repeats [3–5]. The autosomal dominant inheritance of DM1 is accompanied by the phenomenon of anticipation, which is defined as an increased expansion in successive generations accompanied by an earlier and more severe disease course [6]. Specific for DM1 is a severe congenital form. In these patients, first symptoms appear from birth. More frequently however is the adult form with an age of onset in the adolescents. As to date, there is no causal treatment of DM1, thus the therapeutic focus remains on symptomatic treatments [7].

The clinical core pattern of the adult DM1 includes a progressive muscle weakness from distal to proximal and axial combined with myotonic syndrome focused on the hand muscles [8]. Next to various multisystemic symptoms like cataract, jaw and swallow difficulties, gastrointestinal and endocrine involvements, it is the cardiac and respiratory dysfunction defining the severity of this disease [8]. Besides, the variety of cognitive and neurophysiological impairments in DM1 has been frequently described in the past [9–12]. The fatigue syndrome including daytime sleepiness, lack of motivation, difficulty in concentration and unrefreshing naps affects many DM1 patients [10]. These features of myotonic dystrophy often affect the patients’ daily quality of life and restrict the ability for social participating and to work substantially more than the pure muscular symptoms [13]. Researches detected a high incidence of psychiatric co-diagnoses in DM1 patients e.g. attention-deficit/hyperactivity disorder (ADHD), phobia or alexithymia [9]. Recent findings indicated a conspicuous prevalence of autistic spectrum in DM1 [11]. A wide-ranging cognitive retardation was reconnoitred, particularly for congenital and early-onset DM1 patients. Stereotypically, multiple DM1 studies report an IQ level in the lower normal range combined with decreased intellectual abilities [12, 14].

There are only a few studies about dyslexic phenomenon in DM1 patients [15, 16]. The case report by Macniven [16] presents a DM1 patient with progressive dyslexia followed-up of more than 10 years. Dyslexia is a partial performance failure in reading and spelling which is not only caused by a delayed development in cognition or intelligence [17]. Even in the normal population, about 5% of children and adolescents are affected by dyslexia [18, 19]. Chief symptoms of a reading disorder include a significant slow reading speed and consequently difficulties in getting the content right. As the association of individual letters with their corresponding sound is affected, people with reading disorder tend to interchange or skip single letters, especially in complex, polysyllabic and rare words and stressful situations [17]. A spelling disorder presents by a noticeable increased number of spelling errors. Moreover, patients with a spelling disorder try to elude words that probably cannot be spelled correctly to avoid spelling errors [17]. Usually, dyslexia is an impairment of young people, however, adults can be involved as well. Most diagnostic test manuals are not provided for all ages. Only a few standardized tests can be partially used for dyslexia diagnosis in adulthood (e.g. reading disorder: ‘*Salzburger Lese- und Rechtschreibtest’* (SLRT II) [20]). Normative values for the spelling performance in adults do not exist in German test measurements. The comparison of the patients’ test results with the performance of people in the same age, or school year, leads to a percentage ranking (PR). Another important component of the diagnosis of dyslexia consists of the assessment of the intelligence, including the evaluation of logical thoughts and processing speed [17]. Analogous to the diagnosis of dyslexia, treatment options, e.g. reading training, became established especially for children and adolescents however not for adults [21]. Indeed, most important is the clarification of the diagnosis dyslexia to the patient and the social environment to ease the usually tensed situation. A psychotherapy could be indicated as well, as in 40% to 60% dyslexia is associated with psychological problems [17]. In common, it is difficult to find scientific appropriate data on dyslexia in adulthood, as most affected people are children or adolescents.

In patients with DM1, the dyslexic features do not fit into the syndrome of a developmental dyslexia, but rather look like a neurodegenerative process [16]. Brain MRI studies confirm a correlation between progressive cerebral atrophy and cognitive changes, with the focus of white matter lesions in the frontal and parietal lobe in dyslexic persons [22, 23]. As DM1 is known to belong to the spectrum of tauopathies, investigations of the cerebrospinal fluid revealed increased levels of phosphorylated tau protein, which is characteristic for the neurodegenerative disease and dementia [24]. Current studies showed that adult DM1 is accompanied by cognitive decline in memory, attention, visuospatial construction and verbal ability [25].

Our study investigated the prevalence of dyslexia in an adult DM1 cohort by assessing reading and spelling difficulties using a validated test for dyslexia and an IQ test. One core item of our study was the differentiation of a general cognitive retardation to a partial performance failure in the reading performance in DM1 patients to enable a future optimization of diagnostic and therapeutic options.

## Patients and methods

We performed a prospective cohort study inviting adult patients with genetically confirmed DM1, recorded in the *DM-registry of Germany* or the internal DM-database of our neuromuscular department.

The inclusion criteria obtained the following aspects, (1) providing informed consent, (2) age ≥ 18 years, (3) sufficient knowledge of the German language evaluated by two independent investigators.

### ‚Salzburger Lese- und Rechtschreibtest‘ (SLRT II)

For the quantification of the reading performance, we used the validated test measurement SLRT II [20]. The SLRT II is a validated standardized test for the assessment of reading and spelling disorders. According to the SLRT II test manual, especially for adults, the test requirements aim at the detection of deficits in automated, direct word recognition and of synthetic reading, and the detection of weakness in (non-)orthographic writing. Since the letter-to-sound relationship and thus the reading accuracy is usually very high after the first year of school, reading errors are made in the German-speaking area especially, when reading is faster than the reader's abilities allow. The reading accuracy itself, thereby, may not differentiate sufficiently between bad and good readers. On the contrary, the decisive factor is how efficiently, i.e. quickly and fluently, reading is performed. Therefore, reading speed has emerged in recent years as the central criterion for reading performance. Accordingly, the focus of the SLRT II is certainly on the reading fluency and speed, but can nevertheless, based on the above explanation, not only be regarded as a reading fluency task, but as a reading task in general. Reading errors are taken into account to the extent that the number of incorrectly read words is subtracted from the total number. Reading errors and reading time are thus included in a combined value.

In Detail there are two patterns, consisting of 156 words and 156 pseudo-words. Pseudo-words are composed of single letters that do not add up to a familiar word (e.g. ‘mume’). In the first step, the ‘word’ reading test serves primarily to capture automatic, direct word recognition, while, in the second step, the ‘pseudo-word’ reading test serves primarily to capture synthetic reading, i.e. the letter-to-sound relationship. The aim of the test is in the first step to read as many words as possible precisely within one minute, in the second step as many pseudowords. The number of correctly read (pseudo-)words results in a percentage ranking (PR: 0–100). The lower the PR, the worse the reading performance, e.g. if a participant reaches a PR of 20, it means that 80% scored a better test performance than this participant did.

The analysis of spelling errors distinguishes between (non-)orthographical errors (NO-errors) and case sensitivity. N-errors are misspelled words that do not match with the word sound of the test word (for example child – > chid or chilb). O-errors are spellings that match with the sound of the word, but do not represent the grammatically correct spelling (for example, child – > chilt or chilld). In addition, the test detects errors in upper and lower case in the spelling test (case sensitivity). The number of NO-errors results in a percentage ranking (> 2 errors → PR ≤ 80), errors in case sensitivity are based on a critical value of 3.

### CFT-20 R Grundintelligenztest Skala 2‘ in revised ("R") version (CFT 20-R)

To differentiate between dyslexia and a general cognitive impairment, the CFT 20-R was used to assess the IQ [26]. Features as faculty of abstraction, processing speed and logical reasoning are explored by the CFT 20-R. All subtests increase in the level of difficulty. Similar to the SLRT II, the number of correctly solved tasks within a defined time of 3–5 min ends up in an IQ level.

### Diagnosis of dyslexia

The diagnosis of dyslexia in our study was based on the guidelines for diagnosis and therapy of children and adolescents with dyslexia 2015 (S3-guideline) [21]. There are two criteria used in this study provided by the guideline, (1) criterion of the deviation from age level and (2) criterion of IQ deviation. The first criterion is met by patients with a reading performance at least 1.5 standard deviations (SD) below the mean of the same age level. Besides, the guideline presents the criterion of IQ deviation, which can be only used for patients with a below-average reading performance (> 1 SD below normal range). Depending on the patients’ IQ, an appropriate PR is expected for the reading performance. Patients with reading difficulties reaching a PR, which is 1 SD below the expected PR, fit the criterion of IQ divergence. In general, test results of the SLRT II being at least 1 SD below the average can be interpreted as reading difficulties (cutoff value PR ≤ 16, e.g. ≤ 96 correctly read words and ≤ 55 pseudo-words). Further cutoff values are summarized in Table 1.

**Table 1:** Diagnostic criteria for dyslexia due to guidelines for diagnosis and therapy of children and adolescents with dyslexia 2015 (S3-guideline)

Diagnostic criteria for dyslexia		
Diagnostic criteria	Content	Transformation
Criterion of age level divergence	Below-average performance according to the age norm (> 1.5 SD below normal range) in both subtests (pseudo-) word reading	PR < 8 (words) and PR < 11 (pseudo-words)
Criterion of IQ-divergence	Below-average performance in on subtest of the reading performance according to the age norm (> 1 SD below normal range) AND PR in the reading tests 1 SD below the expected PR due to the IQ level	PR ≤ 16 AND Discrepancy > 1 SD between reached PR and expected PR due to the IQ

The data of this study were statistically analyzed with SPSS Statistics 25. For the characterization of the DM1 cohort, the assessment of dyslexic features and for the calculation of reading difficulties, descriptive and explorative testing was used. Further statistical analysis was explored with the *t*-test, comparing the mean values of two independent groups. Normal distribution was determined using the Shapiro–Wilk test and Kolmogorov test. The relationship of non-parametric values was examined by the Spearman correlation.

## Results

### DM1 collective

**Fig. 1:**
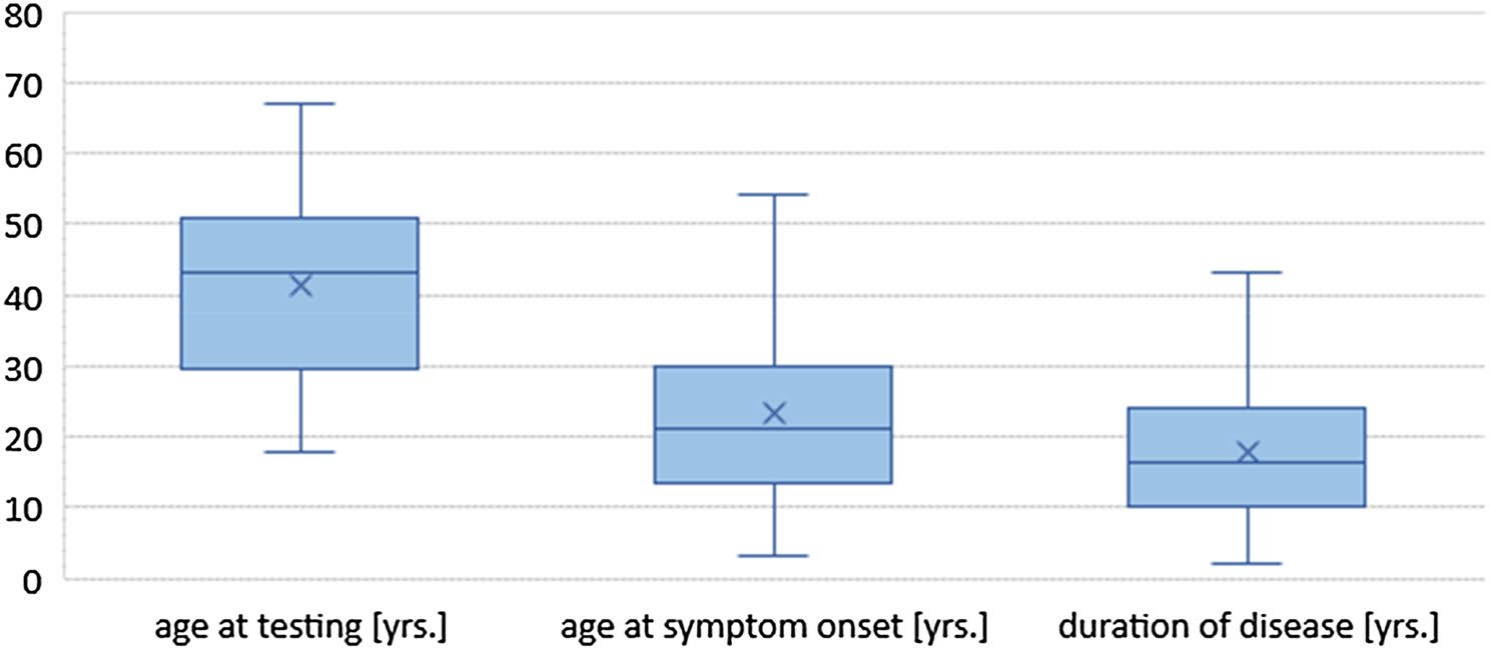
DM1 cohort (*N* = 57) for age at testing, age of onset and duration of disease

Fifty-seven adult patients with genetically confirmed DM1 participated in this study. With a female ratio of 40.4%, the mean age (normal distributed) at testing is 41.4 ± 12.2 years (min–max 18–67; median 43), the mean age of onset is 23.2 ± 13.6 years (min–max 3–54; median 21), the mean duration of disease is 18.0 ± 9.5 years (min–max 2–43; median 16.5) (Fig. 1). The genetic testing of the study participants was not performed at the same time as the assessment of reading and spelling performance but goes back to the year 1989 in single cases. On average, the CTG expansion is 475 ± 332 triplets (min–max 75–1550; median 375; *N* = 55), of these 30.9% < 300, 43.6% between 301 and 600, 16.4% between 601 and 900 and 9.1% > 900 CTG. The muscular impairment of all patients was explored by the muscular impairment rating scale (MIRS): 0 patients MIRS 1; 15 patients (26.3%) MIRS 2; 24 patients (42.1%) MIRS 3; 15 patients (26.3%) MIRS 4; 3 patients (5.3%) MIRS 5.

### Dyslexia

#### Reading disorder

The number of correctly read (pseudo-)words within one minute results in a percentile ranking (PR). 16 DM1 patients (28.1%) of 57 tested study participants had a reading performance 1.5 SD below average values in both, word and pseudo-word reading. These patients meet the criterion of age level divergence (→ *2 Methods*). Further values for reading difficulties and normal reading performances are summarized in Table 2.

**Table 2:** Reading performance due to SLRT II

Reading performance in SLRT II						
	Deviation from normal value	PR word reading	*N* (%)	PR pseudo-word reading	*N* (%)	Intersection *N* (%)
Reading disorder/reading difficulties	> 1 SD	≤ PR 16	23 (40.4)	≤ PR 16	35 (61.4)	22 (38.6)
Norm range	< 1 SD	> PR 16	34 (59.6)	> PR 16	22 (38.6)	21 (36.8)

Those patients that did not meet the criterion of age level divergence were tested for the criterion of IQ-divergence (→ *2 Methods*). Two of these patients had a below-average performance in one reading subtest according to the age norm (> 1 SD below normal range) and a reading performance beneath the PR that was expected due to the IQ value (> 1 SD below expected PR).

**Fig. 2:**
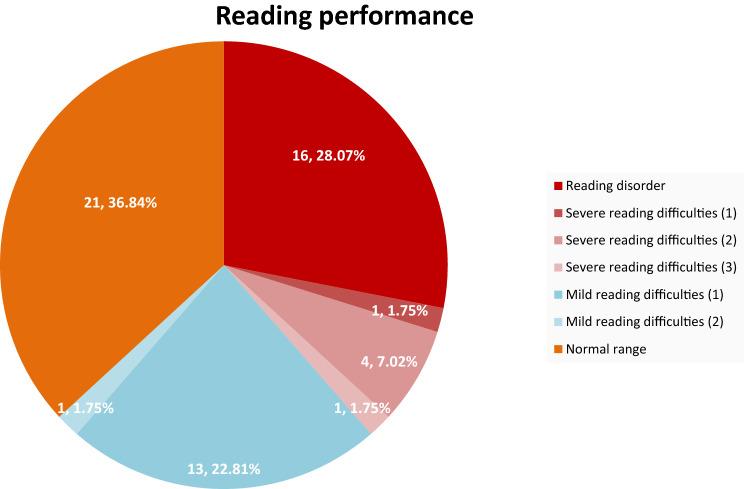
Reading disorder—criterion of age level divergence. *Key notes* Reading disorder: PR [word reading] < 8 and PR [pseudoword reading] < 11. Severe reading difficulties: (1) PR [word reading] < 8 and PR [pseudoword reading] 11–16. (2) PR [word reading] 8–16 and PR [pseudoword reading] < 11. (3) PR [word reading] 8–16 and PR [pseudoword reading] 11–16. Mild reading difficulties: (1) PR [word reading] in normal range and PR [pseudoword reading] ≤ 16. (2) PR [word reading] ≤ 16 and PR [pseudoword reading] in normal range. Normal reading performance: PR [word reading] and PR [pseudoword reading] in normal range (> 16)

Summing up, the prevalence of a reading disorder in the examined DM1 cohort was 31.6% (16 + 2 patients) (Fig. 2). 54.5% of these patients with a reading disorder performing an IQ test reached a normal IQ within 85–115.

There are no significant differences between patients with and without reading disorder according to the age of onset (*p* = 0.160) and duration of disease (*p* = 0.969). A difference was found in regards of the repeat length being significantly increased with 621 ± 100 CTG (median 565 CTG) in patients with reading disorder, compared to DM1 patients without a reading disorder with 409 ± 44 CTG (median 350 CTG) (*p* = 0.027). The gender ratio (female:male) was ≈ 1:5 for DM1 patients with reading disorder, and ≈ 1:1 for DM1 patients without reading disorder.

### Spelling disorder

There exists no appropriate German test manual for the diagnosis of a spelling disorder adapted for adults. Therefore, the criteria of the S3-guideline cannot be transferred to the results of the DM1 cohort in the SLRT II one-by-one, as the reference values for spelling difficulties in this test are only gathered for children.

In summary, in 11 out of 18 patients with a diagnosed reading disorder, at least 3 spelling errors had been observed and therefore correspond to a percentage ranking ≤ 80 that is equivalent to a spelling disorder due to the given reference values for children.

#### Diagnosis of dyslexia (combined reading and spelling disorder)

With reservations, a combined reading and spelling disorder, dyslexia, can be assumed in 11 DM1 patients (19.3%), since the criteria for a reading disorder are fulfilled in 18 DM1 patients, and a spelling disorder can be assumed in 11 of these patients based on reference values for children.

### Individual results of the SLRT II

**Fig. 3:**
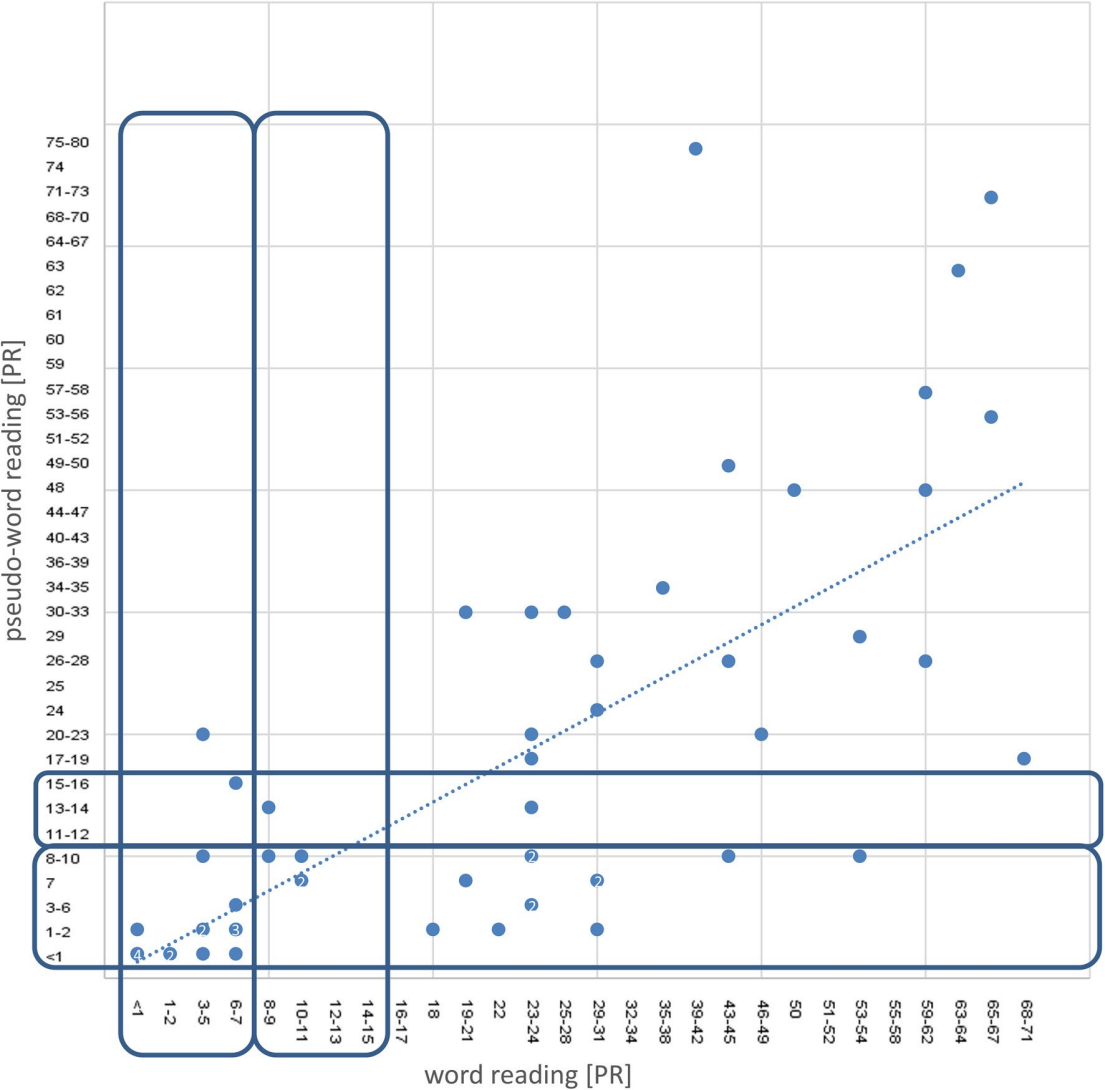
Correlation of word and pseudo-word reading performance in the SLRT II [PR]

In word reading, 40.4% had a below-average reading performance (reading difficulties or reading disorder), in pseudo-word reading there were 61.4% with a reading performance below the normal range (Table 2). On average correctly read words within one minute were 95.0 ± 21.8 (min–max 35–126; median 101; mean PR 12–13) for word reading and 49.4 ± 17.9 (min–max 17–87; median 48; mean PR 8–10) for pseudo-word reading. The PR of word and pseudo-word reading correlates significantly in a moderate degree (*r* = 0.772; *p* = 0.01) (Fig. 3).

**Fig. 4:**
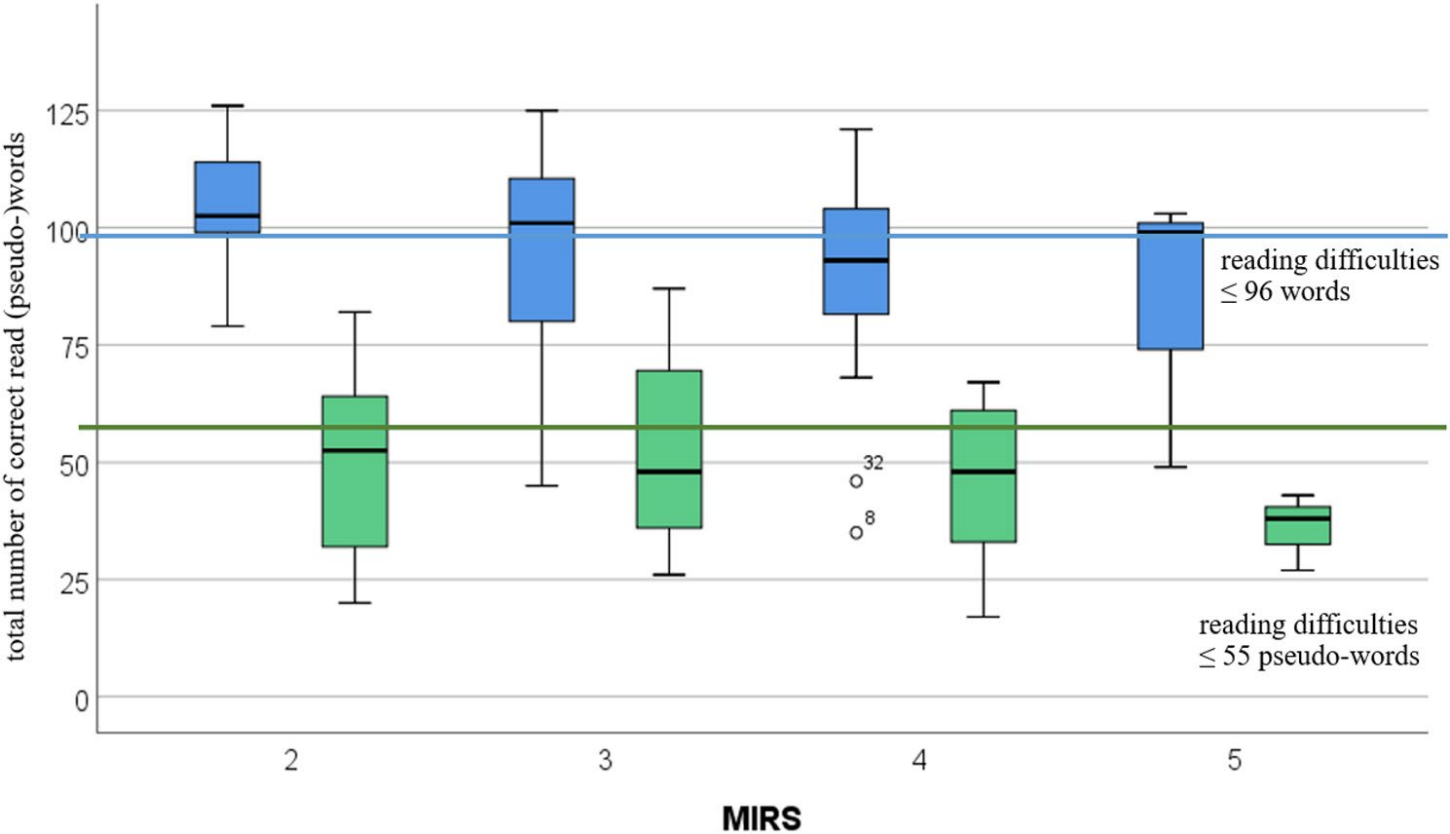
Reading performance—measured by the total number of correct read (pseudo-)words—differentiated by the level of muscle strength—measured by the Muscular Impairment Rating Scale (MIRS) (blue: word reading; green: pseudo-word reading)

The reading performance of the DM1 cohort tends to decline with an increasing MIRS, however not significant in the Spearman correlation (word reading *p* = 0.081; pseudo-word reading *p* = 0.314) (Fig. 4, Table 3).

**Table 3:** Correlation between muscular impairment (MIRS) and reading performance (number of correctly read words)

Correlation between MIRS and reading performance		
	Word reading (*N* ± SD)	Pseudo-word reading (*N* ± SD)
MIRS 1	–	–
MIRS 2	103 ± 14	49 ± 16
MIRS 3	95 ± 23	54 ± 20
MIRS 4	89 ± 24	45 ± 17
MIRS 5	84 ± 30	36 ± 8

### Individual results of the CFT 20-R (IQ value)

The IQ test was performed by 17 DM1 patients. The average IQ was 86.1 ± 19.1 (min–max 54–123; median 82). In total, 41.2% (7 patients) reached an IQ in the normal range [100 ± 15 (1 SD)]. There was a significant difference between the average IQ of patients with maternal (97.9 ± 20.2) and paternal (77.9 ± 13.8) inheritance (*p* = 0.029). The comparison of the average IQ of female and male DM1 patients was inconspicuous (*p* = 0.803).

## Discussion

A general cognitive retardation has been frequently proposed for DM1. Studies deciphering differentiations between a general intelligence impairment and partial performance failures in reading- and spelling performance are rare [8, 15, 18, 27]. Our study focused on the feature of dyslexia and its’ prevalence in an adult DM1 cohort by using a validated test battery for the quantification of the patients’ reading- and spelling performance, and an IQ test for differentiating of a more general cognitive retardation.

The diagnosis of dyslexia is quite complex and based on a multiaxial classification system for mental disorders including a six-axis system. The first axis covers the clinical-psychiatric syndrome, the second refers to the circumscribed developmental deficits, the third to the intelligence level, the fourth to the physical symptoms, the fifth to abnormal associated psychosocial circumstances and the sixth to the global assessment of the psychosocial functional level. The abbreviated testing procedure used in our study represents a screening method, performed by validated and standardized tests. Reading tests validated for the adulthood are rare, spelling problems are even more difficult to explore, as there are no appropriate German tests with cut-off levels. Therefore, the lack of reference values for spelling in adults makes it difficult to make a definitive diagnosis of dyslexia. The SLRT II provides reference values for adults in reading, but only for children in spelling. So all spelling errors made by adult patients examined in our DM1 cohort weigh even more as the test is adapted for children. Using the reference values set for children in an adult collective will probably result in a high false negative number rather than patients with a spelling disorder are recognized as unremarkable. To put it another way, patients with three errors in a test designed for children, would probably perform even worse in a validated test battery for adults. Consequently, the risk of a false positive result of the prevalence of dyslexia in our DM1 cohort is neglectable. With a frequency rate of 19.3% of dyslexia in DM1, this rate clearly exceeds the rate of 5% found in the normal population [18]. Our high prevalence of dyslexia in DM1, determined in this study, stays presently monolithic, as there are no comparable studies available. Nevertheless, a follow-up screening will be of interest exploring the DM1 patients’ reading performance over time.

The study by Macniven presented an unusual long-term case of an adult DM1 patient over 16 years. Part of the assessments was the evaluation of the patient’s reading performance in regular and exception words. At the first reading assessment, 20 years after being diagnosed with DM1, the patient’s reading skills were only mildly impaired on exception words. Throughout the follow-up of the next 10-years, the authors reported a deterioration in both, regular and exception word reading. Similarities to a semantic dementia had been weighed controversially in this report. The observed cognitive decline was associated with a deterioration of the full scale IQ as well [16]. Analogous to our results, the patient’s reading performance was more accurate at reading regular (words) than exception (pseudo-words) words. These results correspond with the observation that unknown words (pseudo-words, exception words) are more difficult to be read correctly, as familiar words can be recognized as total and do not have to be read letter-by-letter. Consequently, people with dyslexic features have difficulties more with pseudo-words than with normal, familiar words.

The comparison of patients with and without a reading disorder according to the age of onset and duration of disease was inconspicuous. Only the variable CTG-repeat length revealed a low significant difference, as dyslexic patients had a longer CTG-repeat expansion on average than non-dyslexics. This correlation between IQ/neurophysiological test results and CTG-repeat length was evident in prior studies [28–30]. Moreover, a trend of a decreasing reading performance with an increasing score on the Muscular Impairment Rating Scale (MIRS) was obvious in our DM1 cohort. The CTG expansion as cofounder for cognitive (reading performance) and muscular impairment (MIRS) could be an explanation for this observed connection. However, the year of the performed genetic diagnosis of our study participants varies between 1989 and 2016, so we do not know the current repeat length at the time of our present testing. The fluctuation of the CTG repeat length over time has not yet been finally clarified [31]. A statement about the correlation between CTG expansion and dyslexic features would be more precise in case of quantifying the reading performance and the genetic testing in the same year. The gender ratio between male and female of 5:1 in the DM1 collective with a reading disorder is consistent with reference values in the normal population [32]. Finally, whether specific RNA splicing events, e.g. of the FOXP2 gene, a gene implicated in speech and language disorders, may contribute to this dyslexic features remain presently open [33].

Another important result to stress was our observation that 54.5% of DM1 patients with diagnosed reading disorder achieved an IQ within the normal range. This emphasizes an independence of dyslexia and IQ. Neither a normal IQ excludes the coincidence of dyslexia, nor a below the average IQ is equivalent to dyslexic difficulties. This finding should have an impact on therapeutic interventions. It is important to realize that people scoring a normal or above-average IQ, nevertheless might need support regarding their dyslexia. Cohen et al. [15] report about children with a high verbal IQ which were benefitting from a special reading training.

Cognitive impairment is a well elaborated finding in congenital and early-onset DM1 patients. Several studies explored the intelligence level for congenital and juvenile DM1, in the majority of cases scoring a below-average IQ [14, 34]. The average IQ in our adult DM1 cohort counts in the lower normal range with 86.1, which is consistent with other study results reporting an average IQ of 82.6 in adult DM1 patients [28]. Moreover, we observed that the average IQ in cases of maternal inheritance exceeds significantly the value of patients with paternal inheritance. Due to the small number of patients, however, this finding needs to be interpreted with caution, since maternal inheritance is more typically associated with the phenomenon of anticipation, and the occurrence of CDM. Considering the average IQ regarding gender differences, there was no difference found between male and female patients. However, a nationwide DM1 cohort reported about a tendency of men being more frequently cognitive impaired than women [35].

Next to some clinical studies that hint at a neurodegenerative cognitive decline in DM1 patients [16], explorations of cerebrospinal fluid detected increased levels of phosphorylated tau proteins, however, with an unclear clinical relevance [24, 36]. Others identified pathological tau protein pattern in DM1 patients that are more consistent with Alzheimer disease [37]. The neurodegenerative process in DM1 has been evaluated in several brain MRI studies. The results are still somewhat inconsistent concerning frequency, extent and significance of the deviations in brain structures. The cerebral changes are focused in the frontal and parietal lobes and consist of volume reduction of grey matter and structural atrophy in the hippocampus [22, 38]. Anomalies in white matter are also reported in DM1, dominating the frontal and temporal lobes [38, 39]. Summing up clinical, histopathological, and imaging results, a neurodegenerative process becomes even more likely. Further studies examining the correlation between clinical behavior and changes in cerebral structures are indispensable, proving a relationship between neuropsychological performance and cerebral tissue modifications [22, 38, 39]. Moreover, long-term follow-ups are needed exploring the neurodegenerative base for DM1 associated dyslexia.

## Conclusion

Our study for the first time elaborated an increased prevalence of dyslexia in adult DM1 patients being independent of the general intelligence. A clinical consequence of our result is an early differential diagnostic assessment of the cognition in DM1. We see the need for distinguishing between a general cognitive impairment and partial performance failure/disorder in reading and spelling. As dyslexia is independent of the general intelligence, it is important not to miss dyslexic features in cognitive inconspicuous DM1 patients. The decision for a detailed diagnosis should be made case-by-case. As therapeutic options and individual support programs are available to support a sufficient education for dyslexic people in school and higher education, an early diagnosis is essential, particularly for children and young adults.

## Data Availability

The data that support the findings of this study are available from the corresponding author, BS, upon reasonable request.
